# Legal hypergraphs

**DOI:** 10.1098/rsta.2023.0141

**Published:** 2024-04-15

**Authors:** Corinna Coupette, Dirk Hartung, Daniel Martin Katz

**Affiliations:** ^1^ Max Planck Institute for Informatics, Saarbrucken, Germany; ^2^ Center for Legal Technology and Data Science, Bucerius Law School, Hamburg, Germany; ^3^ CodeX, The Stanford Center for Legal Informatics, Stanford Law School, Stanford, CA, USA; ^4^ Illinois Tech, Chicago Kent College of Law Chicago, IL, USA

**Keywords:** legal networks, hypergraphs, higher-order networks, temporal networks, complex systems, legal complexity

## Abstract

Complexity science provides a powerful framework for understanding physical, biological and social systems, and network analysis is one of its principal tools. Since many complex systems exhibit multilateral interactions that change over time, in recent years, network scientists have become increasingly interested in modelling and measuring *dynamic* networks featuring *higher-order relations*. At the same time, while network analysis has been more widely adopted to investigate the structure and evolution of law as a complex system, the utility of dynamic higher-order networks in the legal domain has remained largely unexplored. Setting out to change this, we introduce *temporal hypergraphs* as a powerful tool for studying legal network data. Temporal hypergraphs generalize static graphs by (i) allowing any number of nodes to participate in an edge and (ii) permitting nodes or edges to be added, modified or deleted. We describe models and methods to explore *legal hypergraphs* that evolve over time and elucidate their benefits through case studies on legal citation and collaboration networks that change over a period of more than 70 years. Our work demonstrates the potential of dynamic higher-order networks for studying complex legal systems, and it facilitates further advances in legal network analysis.

This article is part of the theme issue ‘A complexity science approach to law and governance’.

## 1. Introduction

In law, myriad entities—e.g. individuals, organizations and texts—interact in intricate ways, producing emergent phenomena, feedback loops and nonlinear change [1]. As such, legal systems are *complex systems* [2–4], and complex systems have long been successfully modelled as *networks* using the language of *graphs* [5].

While considerable progress has been made using simple graphs as models of complex networks, in recent years, the network-science community has embraced more expressive *higher-order network models* [6], allowing us, for example, to distinguish multiple types of nodes and edges (*multilayer networks* [7]) or capture change over time (*temporal networks* [8]). A comparatively recent trend is the systematic study of networks beyond pairwise interactions, allowing interactions between more than two nodes at a time [9]. These networks are naturally modelled as *hypergraphs*, which generalize graphs by allowing edges to contain any nonempty subset of nodes [10]. Acknowledging that n-ary interactions may also evolve over time, we arrive at *temporal hypergraphs* [11–13].

Our work starts from the observation that in the legal domain, interactions constantly change *and* often involve more than two entities [14,15]. For example, in the *legislature* [16,17], lawmakers constantly negotiate new laws, which once passed may repeal or amend old laws, and the composition of the lawmaking body changes with every election. Likewise, in the *judiciary*, individual judges are affiliated with courts and judicial bodies, but they may retire or move between institutions [18], and panels of judges decide cases based on prior decisions, which they often cite in their opinions [19,20]. Hence, legal systems are naturally modelled as *temporal legal hypergraphs*—but as far as we know, this option has not been explored in the literature.^1^

### (a) Contributions

In this work, we make three contributions. First, we introduce *hypergraphs* and *temporal hypergraphs* as mathematical representations of legal network data. Second, we describe classic methods to study temporal hypergraphs in the legal domain. Third, we demonstrate the impact of data-modelling decisions on the output of these methods in case studies on legal information networks and legal collaboration networks, sharing two novel datasets in the process. To the best of our knowledge, we are the first to model legal network data as temporal hypergraphs and highlight the potential of hypergraph methods for understanding legal phenomena.

### (b) Related work

Our work is closely related to two bodies of work: *legal network analysis*, which focuses on our *domain* of interest, and *higher-order network science*, especially the literature studying *hypergraphs*, which engages with our *models* and *methods*.

In *legal network analysis*, inspired by the seminal work of [22], scholars have studied both *information networks*, such as judicial citation networks [19,20,23–26] or legislation networks [14,16,27,28], and *social networks*, such as legislative collaboration networks [29–31] or judicial collaboration networks [32]. While most investigations have focused on individual countries [33–36], others have compared several countries [37,38], or studied networks at the European level [39–42] or the international level [43–46]. Although researchers are increasingly using *temporal graphs* in their modelling [47,48], they have yet to systematically consider higher-order interactions, as has recently been done in other domains [49].

In *higher-order network science*, much work has been done to port concepts and methods from graphs to *hypergraphs*, leading, e.g. to generalizations of walks [50,51], centralities [52,53], motifs [12,54–56], clustering [57–60] and cores [61]. Exploiting edge cardinality as a new source of variation in hypergraphs, researchers are increasingly adapting concepts and methods from *topology* to analyse higher-order network data [13,62,63], and they have also made some progress by leveraging (structurally more constrained) *simplicial complexes* [64–67]. Recent surveys have consolidated our knowledge of when and how to use higher-order network models or collected the mathematical and computational tools currently available for their study [9,68–71], and the software landscape for working with higher-order network data has improved dramatically over the past few years [72–74]. Nevertheless, the study of higher-order network data is still in its relative infancy, especially when compared with traditional network analysis.

### (c) Structure

After describing the two datasets that serve as our running examples in §2, we discuss how these datasets can be modelled as static or temporal (hyper)graphs in §3. In §4, we then introduce different methods for investigating temporal legal hypergraphs and show how these can be used on our datasets, before concluding with a discussion in §5.

## 2. Data

As running examples, we consider two types of network data: judicial decisions and their citations as a classic example of information networks (*legal citation networks*), and arbitrators and their tribunals as a classic example of social networks (*legal collaboration networks*). In particular, we study citation networks derived from the official collection of Germany’s Federal Constitutional Court (GFCC), and collaboration networks derived from the cases registered at the International Center for Settlement of Investment Disputes (ICSID). Notably, these networks also feature different types of temporal evolution: GFCC decisions are associated with one specific date and aggregate over time (*point-aggregation*), and ICSID cases form intervals of collaboration events, associated with a start date and an end date (*interval-event*). Providing a high-level overview of our datasets in table 1, in the following, we give more information on each of our datasets.

**Table 1.** Metadata describing the datasets used as our running examples. We state the network type, the semantics of our raw data along with their count, the temporal coverage of the data along with the number of distinct timestamps *T* in the dataset, and the type of network evolution.

dataset	network type	raw data	cases	coverage	*T*	evolution type
GFCC	citation	case texts	3618	1951–2022 (72 years)	2109	point-aggregation
ICSID	collaboration	case metadata	742	1974–2023 (50 years)	1077	interval-event

### (a) Legal citation networks (GFCC)

We derive our legal citation network data from the texts of judicial decisions published by the Federal Constitutional Court of Germany (*the Court*) in volumes 1–160 of its official collection, BVerfGE. The Court settles questions surrounding the interpretation of the German constitution (*Grundgesetz*), and it is generally known to rely heavily on citations to its own case law to develop its arguments [75]. We source the texts of volumes 1–140 from the online appendix made available by Coupette [19] and collect the texts of the remaining volumes from the Court’s official website (https://www.bundesverfassungsgericht.de/DE/Entscheidungen/Entscheidungen/Amtliche%20Sammlung%20BVerfGE.html).^2^ The result is a *GFCC corpus* of 3618 decisions covering the Court’s most prominent jurisprudence from its inception in 1951 to its latest collection-included decisions rendered in early 2022.

**Figure 1.**
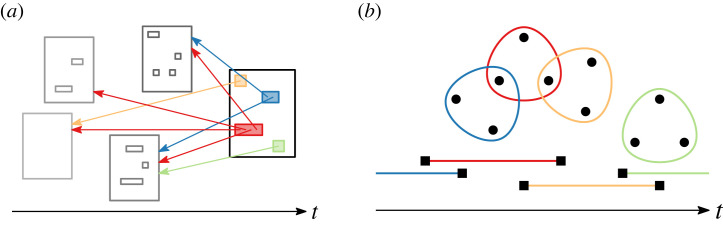
Schematic depiction of the phenomena captured by our datasets. With GFCC, we seek to understand the development of constitutional jurisprudence, with a particular view to how new decisions (large rectangles) create meaning by combining ideas from previous decisions (arrows, drawn here only for the most recent decision, and small rectangles). With ICSID, we would like to explore how the social structure of an elite group, arbitrators (circles) appointed to panels in international-arbitration cases (lines and rounded polygons), evolves over time. (*a*) GFCC, (*b*) ICSID. (Online version in colour.)

For each decision in the GFCC corpus, we extract the other GFCC decisions it cites using a procedure previously introduced in the literature [19]. Notably, our extraction process records the cited decisions at the level of individual *citation blocks*, i.e. uninterrupted strings of cited decisions contained in a citing decision. This allows us to distinguish between decisions cited to illustrate the same legal argument (co-citation in a citation block) and decisions cited (only) in the same decision, providing a more nuanced perspective on the relationships that citations establish between co-cited decisions. We provide a schematic depiction of the phenomenon captured by our GFCC data in figure 1*a*.

### (b) Legal collaboration networks (ICSID)

We derive our legal collaboration network data from the metadata of cases registered at the World Bank’s International Center for Settlement of Investment Disputes (*the Center*) since its establishment in 1966 until mid-June 2023. Among the signatory states of the ICSID Convention, the Center is designed to provide an impartial forum, independent of national courts, for the settlement of investment-related disputes between foreign investors and investee states. All ICSID disputes involve investors as claimants and states as respondents, and they are typically administered by panels of three arbitrators: one appointed by the claimants, one appointed by the respondents, and one jointly appointed by the parties’ selected arbitrators.^3^ We obtain the case metadata from the Center’s website (https://icsid.worldbank.org/cases) and restrict ourselves to cases for which arbitrator names and date information are complete.^4^ This leaves us with 742 cases (corresponding to 2226 tribunal-member slots) registered between 6 March 1974 and 14 June 2023, with participation by 441 unique arbitrators.

In addition to arbitrator names and case-registration dates, we collect and clean the available case metadata on arbitrators’ nationalities, appointing entities, claimants and their nationalities, respondent states, subject and economic sector of the dispute, legal instruments and applicable rules involved, as well as conclusion status, conclusion date, and the date at which the tribunal was constituted. This allows us to analyse several classes of collaboration and affinity relations, including party roles, industry specialization and nationality. A schematic depiction of the phenomenon captured by our ICSID data is given in figure 1*b*.

## 3. Models

**Figure 2.**
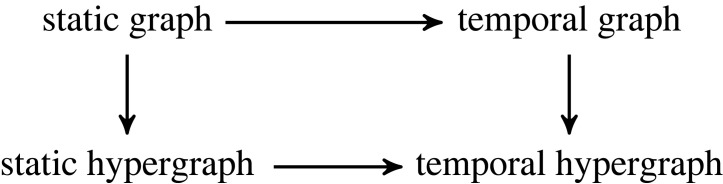
Routes from static graphs to temporal hypergraphs. We will go *via temporal graphs* when modelling the GFCC data, and *via static hypergraphs* when modelling the ICSID data.

Having described our example datasets in §2, we now show how these datasets can be modelled as graphs of varying expressiveness. To this end, we first introduce the relevant abstract concepts, along with their associated notation and basic terminology. We then proceed to model our datasets using these concepts and provide basic statistics of the resulting concrete mathematical objects. As there are two ways to go from static graphs to temporal hypergraphs, depicted in figure 2, in our exposition, we take the route via temporal graphs for the GFCC data and the route via static hypergraphs for the ICSID data.

### (a) Concepts

**Static graphs**. A *simple graph* G=(V,E) is a tuple containing *n* nodes (vertices) $V=\{v_{1},\ldots ,v_{n}\}$ and *m* edges $E=\{e_{1},\ldots ,e_{m}\}$. We call *n* the *order* and *m* the *size* of *G*. If the graph is *undirected*, ei∈(V2) for all i∈[m], where for a set S and a positive integer k≤|S|, (Sk) denotes the set of all k-element subsets of S, and for x∈N with 0∉N, [x]={i∈N∣i≤x}. If the graph is *directed*, $E\subseteq (V\times V)\setminus E_{ii}$, where $E_{ii}=\{(i,i)|i\in V\}$. In *multi-graphs*, edges can occur multiple times, and hence, $E=(e_{1},\ldots ,e_{m})$ is an indexed family of sets, with ei∈(V2) (undirected graphs) resp. $e_{i}\in (V\times V)\setminus E_{ii}$ (directed graphs) for all i∈[m]. We write u∼v if {u,v}∈E in undirected graphs, where *u* is said to be *adjacent* to *v* if u∼v and u→v if (u,v)∈E in directed graphs, where *u* is to be *adjacent* to *v* if u→v or v→u.

In undirected graphs, we denote the *neighbourhood* of node *u* as N(u)={v∣u∼v} and define the *degree* of *u* as δ(u)=|{e∈E∣u∈e}|. In directed graphs, we denote the *out-neighbourhood* of node *u* as $N^{+}(u)=\{v|u\to v\}$, the *in-neighbourhood* of *u* as $N^{-}(u)=\{v|v\to u\}$, and the *neighbourhood* of *u* as $N(u)=N^{+}(u)\cup N^{-}(u)$. Further, we define the *out-degree* of *u* as $\delta^{+}(u)=|\{(i,v)\in E|i=u\}|$, the *in-degree* of *u* as $\delta^{-}(u)=|\{(v,j)\in E|j=u\}|$, and the *degree* of *u* as $\delta (u)=\delta^{+}(u)+\delta^{-}(u)$. Finally, the *line graph* of *G* is L(G)=(E,{{e,f}∣e∩f≠∅}), i.e. each edge in *G* becomes a node in L(G), and two nodes *e* and *f* are connected in L(G) if the edges *e* and *f* intersect in *G*.

**Static hypergraphs**. Generalizing simple undirected graphs, a simple undirected *hypergraph* H=(V,E), also called a *set system*, is a tuple containing *n* nodes V and *m* hyperedges E⊆P(V)∖∅, where P(V)={S∣S⊆V} is the power set of V, i.e. in contrast to ordinary edges, a hyperedge *e* can have any cardinality |e|∈[n]. In an undirected *multi-hypergraph*, $E=(e_{1},\ldots ,e_{m})$ is an indexed family of sets, with $e_{i}\subseteq V$ for all i∈[m]. We define adjacency, node degrees and node neighbourhoods as detailed for simple graphs, and further denote the *edge-neighbourhood* of an edge as N(e)={f∈E∣e≠f∧e∩f≠∅}, as well as its *node-neighbourhood* as $N_{V}(e)=\{v|v\in ⋃N(e)\}$. Observe that while δ(u)=|N(u)| in simple graphs and δ(u)≥|N(u)| in multi-graphs for all u∈V, these relations do not generally hold in hypergraphs. Further, as the cardinality of hyperedges is generally variable, we call a hypergraph *r-uniform* if all hyperedges have the same cardinality, i.e. |e|=r for all e∈E, such that graphs are 2-uniform hypergraphs. As the size of *hyperedge intersections* can vary as well, we can further define an *s-line graph*, which generalizes the line graph by placing edges between two nodes *e* and *f* in L(H) only if |e∩f|≥s.

Lastly, there are two natural *projections* that transform a hypergraph H=(V,E) into a graph: the *bipartite projection*, also known as the *star expansion*, defines $G^{⋆}=(V^{⋆},E^{⋆})$ with $V^{⋆}=V\dot{\cup }E$ and $E^{⋆}=\{\{v,e\}|v\in V,e\in E,v\in e\}$, and it is lossless if we remember the partition of $V^{⋆}$ into the original node and edge sets. The *clique projection*, also known as the *clique expansion*, defines $G^{\circ }=(V^{\circ },E^{\circ })$ with $V^{\circ }=V$ and $E^{\circ }=\{\{u,v\}|\exists \;e\in E:\{u,v\}\subseteq e\}$, i.e. two nodes are adjacent in $G^{\circ }$ if and only if they are adjacent in H. It can optionally be equipped with a *weighting function*, $w:E^{\circ }\to R$ reflecting the intensity of node-to-node associations. The simplest weighting function is $w:E^{\circ }\to N$ with w(e)=|{e∈E∣{u,v}⊆E}| for each $e\in E^{\circ }$, i.e. an edge {u,v} in $E^{\circ }$ is weighted by how often *u* and *v* co-occur in edges from {H}. Even when equipped with a weighting function, clique expansions are generally lossy, i.e. we cannot uniquely reconstruct H from $G^{\circ }$.

**Temporal (hyper)graphs**. There are multiple ways to capture information on temporal evolution in (hyper)graph data. We use the definition of a *snapshot-graph sequence* from Gauvin *et al.* [78], slightly adapted to more naturally accommodate changes to the node set. That is, we define a *temporal graph* $G_{T}=(T,\Gamma )$, where $T=t_{1},\ldots ,t_{T}$ is a sequence of monotonically increasing time stamps, $\Gamma =(\Gamma_{1},\ldots ,\Gamma_{T})$ is a sequence of snapshot graphs $\Gamma_{i}=(V_{i},E_{i})$ for i∈[T], $V_{i}$ is the set of nodes present in graph snapshot i and $E_{i}$ is the set (or indexed family) of edges present in graph snapshot i.^5^ To define a *temporal hypergraph* $H_{T}=(T,\Xi )$, we simply allow hyperedges in $E_{i}$.

### (b) Instantiations

**Legal citation networks (GFCC)**. A legal citation network is classically modelled as a *static directed graph* G=(V,E). In a *judicial* citation network, each node v∈V represents a decision, and each edge e=(u,v)∈E represents a citation u→v. When an edge indicates the *binary observation* that decision *u* cites decision *v* at least once, the result is a *simple* directed graph, whereas when an edge indicates an *observed citation instance*, the result is a directed *multi-graph* (or equivalently, a *count-weighted* directed graph). Observe that judicial decisions (unlike, e.g. statutory texts) cannot typically be altered or removed after they have been published, and that each decision is associated with a decision date. Hence, the graph G=(V,E) used in the static model is simply the last snapshot graph $\Gamma_{T}$ of a *temporal graph* $G_{T}=(T,\Gamma )$ whose time stamps are the ordered sequence of unique decision dates found in the data. Thus, denoting as t(u) the time stamp of decision u, for a snapshot graph $\Gamma_{i}=(V_{i},E_{i})$ with i∈[T], the node set is $V_{i}=\{u\in V|t(u)\leq t_{i}\}$, and the edge sequence is $E_{i}=((u,v)\in E|t(u)\leq t_{i}\wedge t(v)\leq t_{i})$. Note that the resulting snapshot-graph sequence $G_{T}$ is a *growing* graph, i.e. we have $V_{i}\subseteq V_{j}$ and $E_{i}\subseteq E_{j}$ for i,j with $t_{i}\leq t_{j}$.

While temporal graphs retain more information than static graphs, they do not explicitly capture higher-order interactions in legal citation network data. In judicial citation networks in particular, when modelling decisions as nodes and citations as edges, we can only recover which decisions are cited together *in the same decision* by considering the out-neighbourhoods of the nodes (equivalently, we could define a hypergraph in which each citing decision is a hyperedge and each cited decision is a node). However, we lose valuable information regarding which decisions get cited together *in the same legal context*. To capture this information, we can model each decision as a sequence of hyperedges, i.e. $s(u)=(s_{1},\ldots ,s_{c})$, where $s_{i}\subseteq V$ contains the decisions that are cited in context i, and c is the number of distinct contexts in the decision. This requires us to define a *context model* to describe when we deem decisions to be cited in the same context. The practical zeroth-order approach we adopt in this paper is to consider each sequence of consecutive citations (i.e. citations not interrupted by prose) as a separate hyperedge.

**Figure 3.**
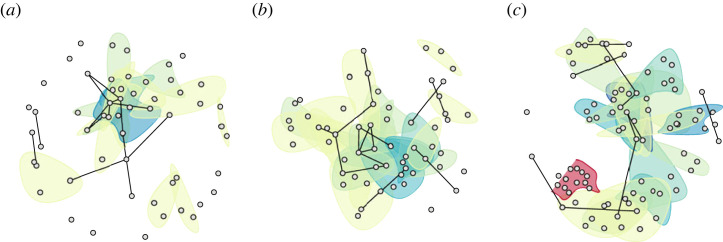
Three prominent decisions from the GFCC corpus depicted as hypergraphs (labeled {volume}, {page}). The decisions concern data privacy (*a*), European integration (*b*) and religious freedom (*c*), respectively. In each panel, nodes represent decisions cited at least once by the visualized decision, and (hyper)edges indicate unique citation blocks. Hyperedge colours progress, by increasing cardinality, from light yellow to dark blue and then red, with binary edges (indicating that exactly two decisions share a citation block) drawn in black. To limit visual clutter, we encode neither the sequential order of hyperedges nor their multiplicities. (*a*) 125, 260—Retention of Data, (*b*) 135, 317—ESM Treaty, (*c*) 153, 1—Headscarf III. (Online version in colour.)

Independent of the context model, the result is a *temporal hypergraph* $H_{T}=(T,\Xi )$ with the same node sets as the temporal graph defined above, and hyperedge sequences corresponding to the contexts that occurred on or before $t_{i}$, i.e. $E_{i}=(*s(u)|t(u)\leq t_{i})$, where we employ ∗ to denote sequence unpacking in a slight abuse of notation. In this framework, individual decisions are represented not only as nodes but also as *(sub-)hypergraphs*, as illustrated in figure 3. Assuming that t(v)≤t(u) for all v∈⋃s(u) (i.e. citations do not point into the future),^6^ just like $G_{T}$ is a growing graph, $H_{T}$ is a growing hypergraph.

**Figure 4.**
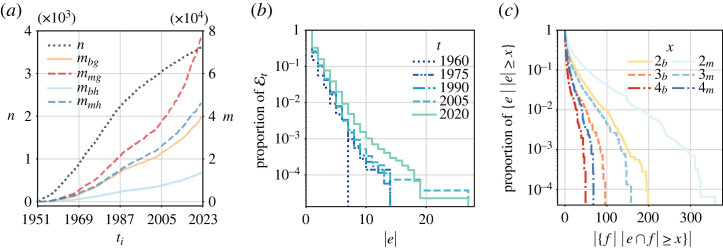
Descriptive statistics based on different network models of the GFCC data. We show (i) the evolution of *n* and *m* in the temporal graph and hypergraph models, (ii) empirical CCDFs of edge sizes at different time points in the temporal multi-hypergraph model (*b*), and (iii) empirical CCDFs of thresholded edge-neighbourhood sizes for the last hypergraph in the temporal hypergraph model with binary edges (b) or multi-edges (m) (*c*). (*a*) Evolution of $G_{T}$ and $H_{T}$, (*b*) edge sizes in $\Xi_{t}$, (*c*) neighbourhood sizes in $\Xi_{T}$. (Online version in colour.)

In table 2a, we provide the basic statistics of the GFCC data when modelled as static (multi-)graphs or (multi-)hypergraphs, supplementing the static description with a temporal perspective in figure 4*a*. We see that the statistics differ widely depending on whether we allow or disallow multi-edges. This indicates considerable redundancy in our data, which can be leveraged to distinguish different intensities of binary or n-ary relationships between the judicial decisions in our corpus. Furthermore, note that under the same edge model, the degree statistics in the graph and the hypergraph are very similar even though the edge counts differ widely—which is a consequence of our definitions. We also see that our median degrees are consistently much smaller than our mean degrees under the same (hyper)graph model. This suggests that our degree distributions are skewed, which can be confirmed by an inspection of the empirical complementary cumulative distribution functions (CCDFs) of node degrees in $\Gamma_{T}\equiv G$. In figure 4*b*, we additionally show the empirical CCDFs of edge sizes at different points in time in our temporal multi-hypergraph model $H_{T}$. Here, we witness a noticeable shift to the right, indicating that citation blocks grow larger over time. Finally, in figure 4*c*, we show the empirical CCDFs of thresholded edge-neighbourhood sizes for hyperedges in $\Xi_{T}\equiv H$, modelled as a simple hypergraph (b) or a multi-hypergraph (m). That is, we ask: for a given edge *e* with |e|=x, how many other edges share at least x nodes with e? We observe that nontrivial nonempty hyperedge intersections (i.e. |e∩f|>1) exist in our data, but also that a large fraction of these intersections is contributed by multi-hyperedges (i.e. citation blocks that occur more than once).

**Table 2.** Basic statistics of our data modelled as static graphs ($\tau_{E}=*g$) or static hypergraphs ($\tau_{E}=*h$), allowing binary edges only ($\tau_{E}=b*$) or permitting multi-edges ($\tau_{E}=m*$). For each (hyper)graph, we state its edge semantics, its number of edges m, as well as its mean degree $\bar{\delta }$ and its median degree $\delta_{M}$. For the directed GFCC graphs, we report the average out-degree (equal to the average in-degree) under $\bar{\delta }$, and separate values for the median out- and in-degrees under $\delta_{\text{M}}$.

(a) GFCC					(b) ICSID				
edges	$\tau_{E}$	*m*	$\bar{\delta }$	$\delta_{\text{M}}$	edges	$\tau_{E}$	*m*	$\bar{\delta }$	$\delta_{\text{M}}$
citations	bg	39 428	10.9	$6^{+}|7^{-}$	case sharing	bg	1 869	8.5	4
	mg	77 284	21.4	$9^{+}|10^{-}$		mg	2 226	10.1	4
citation blocks	bh	13 541	10.2	6	shared cases	bh	722	4.9	2
	mh	46 257	21.4	10		mh	742	5.0	2

**Legal collaboration networks (ICSID)**. A legal collaboration network is classically modelled as a *static undirected graph* G=(V,E). In an *arbitrator* collaboration network, each node v∈V represents an arbitrator sitting on at least one tribunal, and each edge e={u,v}∈E indicates that arbitrators *u* and *v* share a tribunal. The result is an undirected *simple* graph when an edge models the *binary observation* that *u* and *v* sat on the same tribunal at least once, and an undirected *multi-graph* (or equivalently, an undirected *count-weighted* graph) when an edge models an *observed collaboration instance*. In this modelling framework, every ICSID case induces three (potential) edges, one between each pair of arbitrators that is part of the case tribunal. That is, the static graph *G* is a *clique expansion* of the static 3-uniform hypergraph H=(V,E) with the same node set as G, and the edge sequence containing one hyperedge for each constituted tribunal of a case.

While moving from static graphs to static hypergraphs allows us to investigate higher-order interactions in ICSID arbitration tribunals, we have yet to account for temporal evolution. To this end, observe that unlike the citations in the GFCC data, which enter the citation network and then stay there, collaborations on ICSID tribunals only exist while the case is active. We proxy the activity timespan of a case by the closed interval between its tribunal constitution date and its conclusion date. Therefore, when we model the ICSID data as a temporal hypergraph $H_{T}=(T,\Xi )$, our time stamps are the ordered sequence of unique dates on which a tribunal is constituted *or* a case is closed. Denoting as $t_{\min}(e)$ the tribunal constitution date of a case e, and as $t_{\max}(e)$ its conclusion date, for a snapshot hypergraph $\Xi_{i}=(V_{i},E_{i})$ with i∈[T], our edge sequence is $E_{i}=(e|t_{\min}(e)\leq t_{i}\wedge t_{\max}(e)\geq t_{i})$, and our node set is $V_{i}=\underset{e\in E_{i}}{⋃}e$. This construction implies that δ(v)>0 for all $v\in V_{i}$. In contrast to our GFCC data, due to the interval nature of ICSID collaborations, the temporal hypergraph derived from our ICSID data is *not* growing.

**Figure 5.**
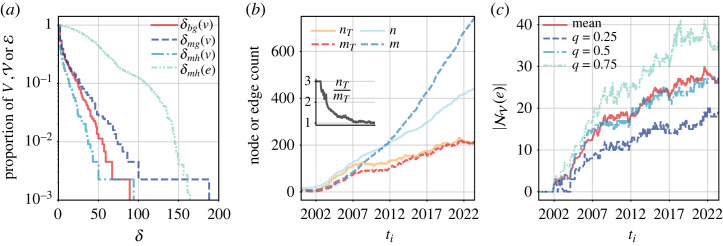
Descriptive statistics based on different network models of the ICSID data. We show (i) the empirical CCDFs of degrees for the static graph *G* and the static hypergraph *H* (*a*), (ii) the evolution of *n* and *m* in $H_{T}$ in contrast to a static *H* cut off at the same time stamp (*b*), and (iii) statistics characterizing how the distribution of the number of nodes (arbitrators) in the neighbourhood of an edge (case) in $H_{T}$ changes over time (*c*). (*a*) Degrees in *G* and *H*, (*b*) order and size in $H_{T}$ and H, (*c*) neighbourhood sizes in $H_{T}$. (Online version in colour.)

In table 2b, we provide the basic statistics of the ICSID data when modelled as static (multi-)graphs or (multi-)hypergraphs. We see that in the representations allowing multi-edges, the graph representation has exactly three times as many edges as the hypergraph representation, and its average and median degrees are twice as high. This is a consequence of the fact that *H* is 3-uniform and *G* is a clique expansion of *H*. Furthermore, the differences between the edge counts of our binary and multi-edge models show that while there is a considerable fraction of repeated *collaboration pairs* ($\frac{357}{2\;226}=0.16$), the fraction of repeated *collaboration trios* is much smaller ($\frac{20}{742}=0.03$)—but both fractions are significantly higher than expected under a random model.^7^ Similar to our findings for the GFCC data, we again observe median degrees that are much smaller than mean degrees, with $\bar{\delta }>2\delta_{\text{M}}$ in all network models, and as illustrated in figure 5*a*, the degree distributions are skewed, too.^8^ To demonstrate the impact of temporal modelling on our ICSID data, in figure 5*b*, we show the order and size of hypergraphs in $H_{T}$ as compared to those of a static hypergraph *H* that, at time $t_{i}$, includes all cases whose tribunal was constituted on a date no later than $t_{i}$ (effectively following a point-aggregation instead of an interval-event evolution model). We see that the total number of cases (*m*) grows about twice as fast as the number of active cases ($m_{T}$), and the total number of arbitrators (*n*) grows more than three times as fast as the number of active arbitrators ($n_{T}$). Notably, the number of active arbitrators per active case ($n_{T}/m_{T}$) drops from 3 in the early 2000s to around 1 in mid-2023 (i.e. on average, each active case shares two arbitrators on its tribunal with other active cases), indicating increasing concentration of cases among arbitrators. This concentration is also reflected in figure 5*c*, where we depict the temporal evolution of point statistics showing how many arbitrators are at most one hop away from an individual case (i.e. $|N_{V}(e)|$ for $e\in E_{i}$ at time stamp i). Figure 5*c* further reveals that the mean and median node-neighbourhood sizes of edges remain consistently close to each other, which contrasts with the discrepancy between mean and median node degrees recorded in table 2b. Interestingly, while the mean node-neighbourhood size of edges has roughly doubled between 2007 and 2023, with an active case now having more than 25 arbitrators in its immediate vicinity, we also find that although over 90% of nodes have been part of the largest connected component, the diameter of this component has remained relatively large (i.e. between 6 and 9).

## 4. Methods

In §3, we found that the representation choice, and especially the modelling decisions (i) *graph* or *hypergraph*, (ii) *static* or *temporal* and (iii) *binary* edges or *multi*-edges, can heavily influence our results when we analyse legal citation or legal collaboration network data—at least if we look at basic statistics. Now, we investigate whether this also holds when applying established network-analysis methods. Starting from the micro level and gradually zooming out to the meso level, we demonstrate how generalizations of three classic network-analysis concepts can be applied to (temporal) legal hypergraphs: *centralities*, *motifs* and *communities*.

### (a) Centralities

*Centralities* are fundamental tools for assessing the *importance* of individual nodes or edges in a graph, whether importance is considered to mean sheer connectedness (*degree*), the ability to quickly disseminate information (*closeness*) or gatekeeping power (*betweenness*). A number of centrality measures have been generalized from graphs to hypergraphs, including the aforementioned centralities [50] as well as eigenvector centralities [52]. Since hyperedges can intersect in more than one node, walk-based centrality measures can additionally be parametrized by a required intersection size s, which determines how many nodes must be shared between two hyperedges for them to be considered connected in the line graph L(H). Furthermore, *hyperedge* centralities play a more prominent role in hypergraph analysis than *edge* centralities in classic network analysis.

**Figure 6.**
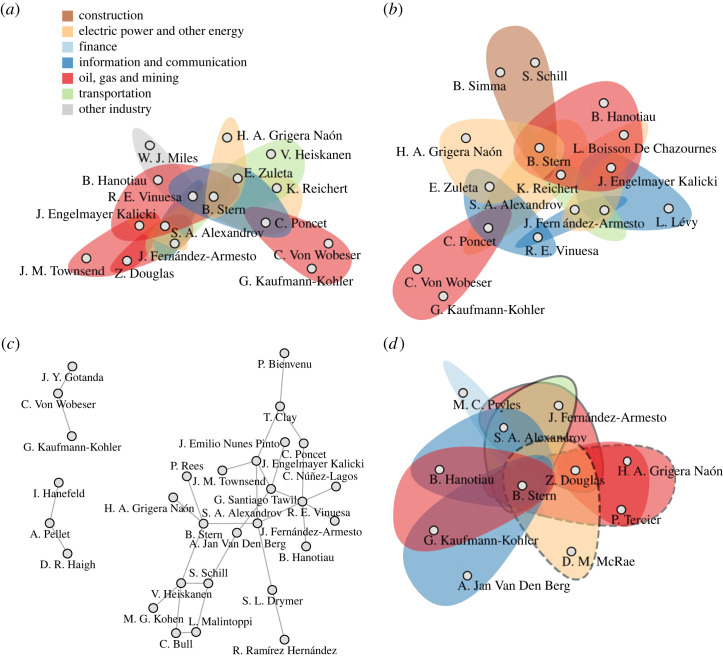
Centrality backbone of the ICSID data as assessed by different centrality measures. Hyperedges represent ICSID cases and nodes represent arbitrators participating in their tribunals. We show the top-ranked hyperedges based on the last snapshot of the *temporal* hypergraph representation, $\Xi_{T}$, using either hyperedge 1-betweenness centrality ((*a*), hyperedges ranked ≤10) or hyperedge 1-closeness centrality ((*b*), hyperedges ranked ≤10), the top-ranked edges (indicating that two arbitrators shared a tribunal) based on the last snapshot of the temporal *graph* representation, $\Gamma_{T}$, as judged by edge betweenness centrality ((*c*), edges ranked ≤30), and the top-ranked hyperedges based on the *static* hypergraph representation, H, as judged by hyperedge 1-closeness centrality ((*d*), hyperedges ranked ≤7). In (*a*,*b*,*d*), cases are coloured by their economic sector. In figure 6*d*, we additionally surround cases concluded *before* 2017 with dashed black lines and cases whose tribunal was constituted *after* 2017 with solid black lines, thus highlighting that not all top-ranked cases in *H* had overlapping activity times. (*a*) Hyperedge 1-betweenness centrality on $\Xi_{T}$. (*b*) Hyperedge 1-closeness centrality on $\Xi_{T}$. (*c*) Edge betweenness centrality on $\Gamma_{T}$. (*d*) Hyperedge 1-closeness centrality on *H*. (Online version in colour.)

**ICSID example**. To illustrate the impact of temporal (hyper)graph modelling on micro-level centrality assessment, in figure 6, we show the highest-centrality edges—informally termed the *centrality backbone*—of the ICSID data, modelled as a temporal hypergraph, a temporal graph, or a static hypergraph, as judged by either shortest-path betweenness centrality or shortest-path closeness centrality. Here, edge betweenness is the classic shortest-path betweenness centrality for edges, hyperedge betweenness is *1-betweenness centrality*, i.e. shortest-path betweenness centrality of nodes in the 1-line graph associated with the hypergraph, and hyperedge closeness is *1-closeness centrality*, i.e. shortest-path closeness centrality of nodes in the 1-line graph.^9^ We see that the backbone obtained from the temporal graph representation does not contain any triangles, i.e. it does not respect the *higher-order* structure of the data. Similarly, the backbone obtained from the static hypergraph representation contains cases that overlap in their tribunal members but not in time, i.e. it ignores the *temporal* structure of the data.

### (b) Motifs

*Motifs* are small, connected, non-isomorphic subgraphs that are statistically overrepresented in an observed graph when compared with some randomized null model [79]. This idea straightforwardly translates to the hypergraph setting, with the modifications that (i) k-motifs can contain hyperedges of cardinality up to k, and (ii) the null model needs to be a randomized *hypergraph* model. Here, the simplest choice is the *hypergraph configuration model*, which keeps the node-degree and hyperedge-cardinality distributions and randomizes node-to-hyperedge affiliations [56,80].

**Figure 7.**
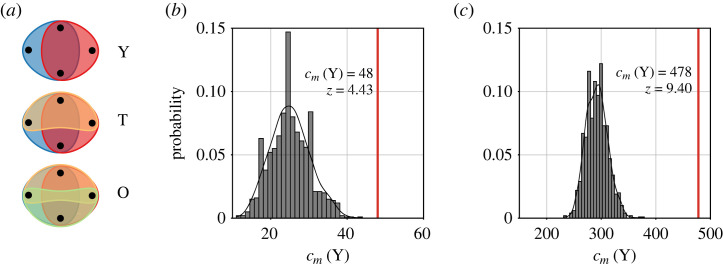
Theoretically possible 4-motifs in 3-uniform hypergraphs (*a*), and frequency of the Y-motif in a population of 1000 hypergraphs drawn from the configuration model, with the same node-degree distribution and edge-cardinality distribution as our ICSID hypergraph (red line), for the last hypergraph snapshot in our temporal hypergraph $\Xi_{T}$ (*b*) and the statically modelled hypergraph *H* (*c*), with annotated counts $c_{m}(Y)$ and z-scores z. In the ICSID data, the Y-motif occurs much more frequently than expected under the null model. (*a*) Feasible 4-motifs. (*b*) Y-motif in $\Xi_{T}$. (*c*) Y-motif in *H*. (Online version in colour.)

**ICSID example**. To show how temporal (hyper)graph modelling impacts meso-level motif analysis, we again focus on the ICSID data. Recall that our ICSID hypergraphs are 3-uniform, such that there are only three 4-motifs, displayed in figure 7*a*, that could theoretically occur in our data. Calling these motifs *Y*, *T* and *O* to reflect their shape, we count how often each of these potential motifs occurs, both for individual snapshot-hypergraphs of the temporal hypergraph $H_{T}$, and for the static hypergraph *H* that aggregates all case observations.^10^ We then compare the empirical counts with the counts we observe in 1000 hypergraphs drawn from a hypergraph configuration model. The results, depicted in figure 7*b* and 7*c* for the Y-motif in $\Xi_{T}$ and in *H*, indicate that we observe far more case pairs that share two arbitrators than we would expect if active arbitrators were assigned to cases randomly (figure 7*b*), and aggregating cases over time only makes the discrepancy between the observed hypergraph and the null model more extreme (figure 7*c*). While we do not observe the O-motif—either in the ICSID data or in the hypergraphs sampled from the configuration model—we *do* observe individual occurrences of the T-motif in some snapshot hypergraphs, and seven occurrences of the T-motif in the aggregated, static *H* (z=6.95). This is remarkable because the T-motif is not compatible with complete *role specialization* among arbitrators: with three cases each sharing two arbitrators, it is impossible to assign the roles president, claimant and respondent to arbitrators such that each arbitrator has the same role in all cases.

### (c) Communities

Moving beyond the local structures captured by motifs, identifying *communities* (or *clusters*) of closely related nodes is one of the most important meso-level tasks in network analysis [81]. While some hypergraph-specific clustering methods exist, many of them implicitly work with a projection onto some kind of graph [82], and the toolkit for community detection in graphs is currently much more flexible. Thus, we would like to encode the information contained in a legal hypergraph *H* in a *weighted graph* $G_{w}$ that can be used as an input to existing community-detection algorithms. How exactly this translation should proceed depends on the semantics of the underlying data. Hence, our goal here is merely to illustrate one plausible approach, tailored to judicial citation data, and to demonstrate that this approach works well in practice.

To see how we can transform a hypergraph representing legal citation data into a weighted graph reflecting its higher-order relationships, we focus on data that are structured like our GFCC data. Recall that in these data, nodes correspond to judicial decisions and hyperedges correspond to citation blocks, which are themselves associated with the decision in which they occur (i.e. we can think of citing decisions as *hyperedge colours*). Therefore, a citation block provides evidence for pairwise associations between the decisions contained in the block as well as between the citing decision and each of the cited decisions, and we would like to define the edge weights of our weighted graph to reflect the strength of this evidence. We make the following assumptions: (i) For associations between co-cited decisions, the smaller the hyperedge, the stronger the evidence.^11^ (ii) For associations between the citing decision and the co-cited decisions, if we want to include them at all, the more a decision gets cited and the fewer other decisions get cited, the stronger the evidence. Hence, we begin by defining $G_{w}=G^{\circ }=(V^{\circ },E^{\circ })=(V,\{\{u,v\}|\exists \;e\in E:\{u,v\}\subseteq e\})$, i.e. the skeleton of $G_{w}$ is the clique expansion of H=(V,E). We then define $w:E^{\circ }\to R$ with

$$w(\{u,v\})=\sum_{\{u,v\}\subseteq e}\frac{1}{|e|-1},$$

i.e. for each hyperedge *e* of cardinality at least 2 in *H*, we add 1/|e|−1 to the weight of each edge {u,v} with {u,v}⊆e. This implies that the sum of the weights added to the edges incident with node *u* in $G_{w}$ as a consequence of its membership in a hyperedge *e* is exactly 1. It also ensures that a random walk on the resulting graph that picks its next destination proportionally to the edge weights corresponds to a non-lazy random walk on the hypergraph *H* that, when at node u, first picks a hyperedge *e* uniformly at random and then picks a node v∈e∖{u} uniformly at random (this is called an *equal-edges random walk* in [62]).

**Figure 8.**
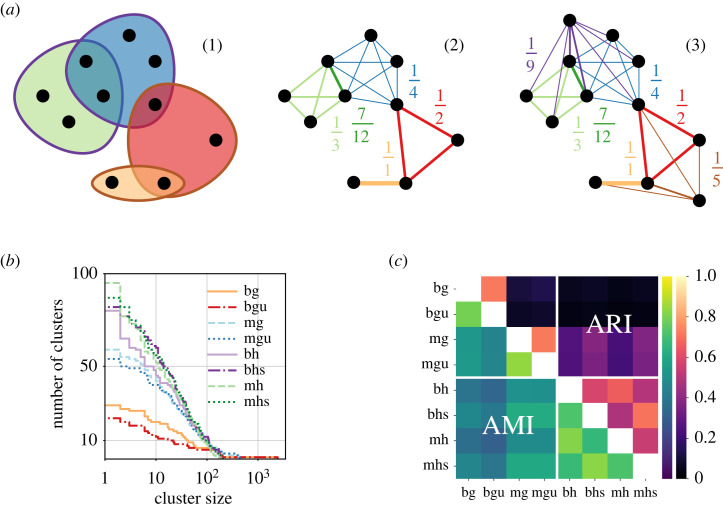
In the association-graph construction, we transform (1) a hypergraph *H* with coloured hyperedges (indicating citation blocks that belong to the same decision) into (2) a weighted clique expansion of *H*, optionally (3) augmenting this expansion with star-expansion-like self-affiliations (*a*). *Infomap* clusterings are more balanced when based on an association-graph representation derived from the GFCC hypergraph than when based on one of the traditional directed graph representations, as indicated by the cluster-size distribution of the AMI-determined medoids of 50 differently seeded clusterings using the same model (*b*). Clusterings based on association graphs also capture node-to-node relationships differently, as indicated by the AMI and ARI scores of pairwise comparisons between the medoid clusterings of each model (*c*). In figures (*b*,*c*), labels encode the underlying (hyper)graph representation as (*b*inary∣*m*ulti)(*g*raph∣*h*ypergraph)[*s*elf-association (versus none)∣*u*ndirected flow (versus directed)]. (*a*) Association-graph construction, (*b*) medoid cluster-size distribution. (*c*) medoid clustering similarity. (Online version in colour.)

Note that $G_{w}$ as defined above will contain relatively little information about recent decisions, as these decisions did not get many opportunities to be cited by other decisions. To enable clustering algorithms to also integrate these decisions, we can opt to further include the evidence provided by the *self-association* of all decisions in the form of their own citations (at the expense of a *clean* correspondence between a random walk on *H* and a random walk on $G_{w}$). That is, we can add all citing decisions to $V^{\circ }$, and for a decision *u* containing citation blocks $E_{u}$, to each edge {u,v} with $v\in E_{u}$, we can add the weight $\frac{|\{e|v\in e\wedge e\in E_{u}\}|}{\sum_{e\in E_{u}}|e|}$, i.e. the fraction of citations by *u* that go to v, which implies that the sum of the weights added to edges by the self-association of *u* is exactly 1. Illustrating the construction in figure 8*a*, we call the resulting graph, which is a hybrid between a weighted clique expansion and a weighted star expansion, the *association graph* of *H*.

**GFCC Example**. To see how using an association-graph representation instead of a classic graph representation impacts clustering results, we compute 50 clusterings for each of eight different GFCC data representations, four hypergraph-derived association-graph representations and four traditional graph representations. We use *Infomap* [83] as our clustering algorithm because (i) its theoretical foundation based on random walks mimics the legal search process, (ii) it does not require us to specify the number of communities but rather derives it from the data based on information-theoretic principles and (iii) it has been shown to work well on classic representations of legal citation data [14,19,32]. Moreover, the group developing *Infomap* has tested the algorithm for hypergraph clustering based on clique expansions similar to those defined above [82]. Evaluating our clustering results is not straightforward, especially because (i) our clusterings are based on *different* (hyper)graph representations of the same data, (ii) there is no universally accepted *ground truth* and (iii) unsupervised quality measures do not necessarily capture clustering quality from a *legal* perspective. To understand how our clustering results are related and gauge clustering quality, we thus combine objective quantitative measures and subjective qualitative assessments.

We begin by investigating the quantitative structure of the partitions associated with each clustering. To this end, in figure 8*b*, we show the cluster-size distribution of the *medoid* of 50 differently seeded clusterings for each of our eight (hyper)graph representations. Here, the *medoid* of a set of clusterings using the same underlying representation is the clustering with the largest sum of within-model adjusted mutual information (AMI) scores, indicating that on average, it is closest to all other clusterings using the same model (at least when judged by AMI). We observe a steep step from cluster size 1 to larger cluster sizes in both association-graph representations not using self-affiliation (*bh* and *mh*), which is absent in the results using self-affiliation (*bhs* and *mhs*), demonstrating the importance of including self-affiliation when working with the hypergraph-based model. Furthermore, we see that clusterings based on association-graph representations tend to be rather balanced, with the three largest clusters each holding between 4% and 6% of all nodes, whereas clusterings based on classic graph representations (*bg*, *bgu*, *mg*, *mgu*) tend to have one to three large dominant clusters containing between 10% and 70% of all nodes. Hence, from a *balancedness* perspective, clusterings leveraging affiliation-graph representations with self-affiliation (*bhs*, *mhs*) appear to be preferable over all other clusterings. Notably, clusterings based on affiliation-graph representations also achieve *consistently* higher *performance*^12^ than those based on classic graph representations—i.e. the former exhibit higher performance than the latter *even* when both are evaluated on *classic* graph representations.

To further explore the relationships between our clusterings, in figure 8*c*, we show the pairwise similarities between the medoid clusterings, as assessed by their AMI scores and their adjusted rand index (ARI) scores. We see that the similarities between clusterings based on association-graph representations on the one hand and clusterings based on classic graph representations on the other hand are relatively low (upper right and lower left quadrant), which suggests that association graphs capture node-to-node relationships differently from classic graph representations. However, this can also be said, more generally, for each pair of representations using dissimilar underlying models, as outside the submatrices {{*bg*, *bgu*}, {*mg*, *mgu*}, {*bh*, *mh*}, {*bhs*, *mhs*}}, both AMI and ARI are relatively low. Notably, the scores also reflect the large variation in cluster sizes between the different medoid clusterings (cf. figure 8*b*).

Lastly, we *manually* inspect our medoid clusterings, assigning labels to their individual clusters based on the titles (or rather: taglines) of the clustered decisions provided by the Court (available in the online materials). Here, we focus on the most promising representations based on the affiliation graph and the classic graph, namely, *mhs* and *mbu*. We find that while both clusterings capture the high-level structure of German Federal Constitutional Court jurisprudence, the hypergraph-based clustering tends to provide a more fine-grained map of German constitutional law. As a result, we can observe the evolution of distinct lines of case law at a higher level of detail, unearthing, for example, a sequence of decisions on the legal status of Berlin as a special case in a more general line of decisions on state-to-state and state-to-federation relations.

## 5. Conclusion

In this work, we proposed *hypergraphs* and *temporal hypergraphs* as mathematical representations of legal network data. We showcased how generalizations of established network-analysis methods can be used to study temporal hypergraphs in the legal domain, and we demonstrated how data modelling decisions impact the results of these methods in case studies on legal citation networks (GFCC data: citations in German Federal Constitutional Court decisions) and legal collaboration networks (ICSID data: arbitration tribunals of cases at the International Center for Settlement of Investment Disputes). While we provided some evidence for the potential of temporal legal hypergraphs as representations of legal network data, we have arguably only scratched the surface—especially in terms of our data, methods and applications. Therefore, we see avenues for future work in several directions. First, regarding *data*, it would be interesting to see existing legal network datasets modelled as temporal hypergraphs, and the results of prior legal network studies could be re-evaluated based on the resulting representations. Second, regarding *methods*, we see great opportunities in translating further concepts from graphs to hypergraphs, and in exploring novel approaches to hypergraph data analysis based on representations of hypergraphs as filtered simplicial complexes or partially ordered sets—which will allow us to better understand, inter alia, the evolution of legal argumentation. And third, regarding *applications*, we are curious to see what further insights in-depth analyses of temporal legal hypergraphs can reveal about the structure and dynamics of complex legal systems.

## Data Availability

All data, code and results are available at the following DOIs: (i) reproducibility package: https://doi.org/10.5281/zenodo.8081507 [84], (ii) GFCC data: https://doi.org/10.5281/zenodo.8081511 [85] and (iii) ICSID data: https://doi.org/10.5281/zenodo.8081513 [86].

## Footnotes

1 The only (partial) exception we are aware of is the work by Boulet *et al.* [21], who model sequences of multilateral-treaty ratifications as directed hypergraphs, encoding time in hyperedge directions (based on a non-standard definition of directed hypergraphs). However, they only use the incidence matrix of this hypergraph to embed their nodes (representing countries) in $R^{k}$ for some chosen (undisclosed) k and then use methods tailored to point-cloud data to perform the rest of their analyses.

2 Although the Court provides an overview of the decisions included in its official collection on its website, its documentation is still incomplete (e.g. some decisions included in the collection are missing from the overview) and the text of most older decisions is not currently available from the website.

3 The procedural details and arbitration realities are more complicated. For a review of the rules governing ICSID’s operations, including the selection of arbitrators, see [76]. For a summary of concerns surrounding ICSID’s operations, see [77].

4 By completeness of *arbitrator names*, we mean that we can identify one arbitrator in each role. We do not currently account for changes in panel composition while a case is ongoing (identified arbitrators correspond to the *latest* entries in the ICSID database), which will be of interest to scholars specializing in international arbitration. By completeness of *date information*, we mean that (i) the case is either still pending or we know when it was concluded and (ii) the tribunal constitution date is available. For all cases fulfilling these conditions, a case registration date is also available (which usually, but not always, lies before the tribunal constitution date), but there are three cases that fulfil condition (i) for which we know only the registration date but not the tribunal constitution date. For consistency, we exclude those cases from our analysis.

5 The difference to the definition from Gauvin *et al.* [78] is that we allow the node sets to differ between snapshots.

6 Our GFCC dataset contains 38 instances of citations forward in time and 55 instances of citations where source decision and target decision were rendered on the same date. While forward citations mostly result from editorial choices concerning the collection, lateral citations can also follow from scheduling decisions within the Court.

7 For 1000 draws from the hypergraph configuration model (see §4), the z-score for observing 357 redundant collaboration *pairs* is 4.81, and the z-score for observing 20 redundant collaboration *trios* is 10.40.

8 Much of the skew in node degree distributions is due to one arbitrator, *Brigitte Stern*, who sat on the tribunals of 94 cases in our corpus, serving as the president in 2 cases, and appointed by (or on behalf of) respondents in the remaining 92 cases.

9 Note that temporality is reflected in figure 6*a*,*b* via the *structure* of the underlying hypergraph: Only cases active in the last snapshot of $H_{T}$ are considered present for the purposes of our centrality assessment. This contrasts with the *aggregated* input data underlying figure 6*d*, which was never observed as a snapshot (and hence, should *not* be analysed using a static centrality measure). Developing *temporal* centrality measures that operate on the *entire* $H_{T}$ presents a great opportunity for future work.

10 In the terminology of Gauvin *et al.* [78], we randomize both hypergraphs using degree-constrained link shufflings (based on their implicit link-timeline representations). Note that for the purposes of this exposition, we focus on how data-modelling decisions concerning our input data impact our results. Hence, we do not require motifs to be temporally connected (e.g. a Y-motif can consist of two cases that share two arbitrators, even if these cases are disjoint in time).

11 This assumption is *self-contained*, i.e. it does not require any knowledge regarding the relationship between co-cited decisions that is not expressed in the data. While testing it empirically could yield an interesting contribution to the study of judicial citation practice, our theoretical justification of this assumption is that (1) one function of citation blocks is to back up contentious points, (2) the more contentious a point, the more supporting evidence should be provided for an argument to be convincing and (3) the more decisions are cited together, the fewer features will be naturally shared by *all* of them. Alternative assumptions, e.g. that each co-occurrence of two decisions in a citation block provides the same level of evidence (which would also be self-contained), can easily be incorporated by adjusting the weight-allocation procedure of our graph $G_{w}$.

12 *Performance* is defined as the number of intra-community edges plus the number of inter-community non-edges, divided by the total number of potential edges.
